# Surface Defect Detection for Small Samples of Particleboard Based on Improved Proximal Policy Optimization

**DOI:** 10.3390/s25082541

**Published:** 2025-04-17

**Authors:** Haifei Xia, Haiyan Zhou, Mingao Zhang, Qingyi Zhang, Chenlong Fan, Yutu Yang, Shuang Xi, Ying Liu

**Affiliations:** 1 Jiangsu Co-Innovation Center of Efficient Processing and Utilization of Forest Resources, College of Mechanical and Electronic Engineering, Nanjing Forestry University, Nanjing 210037, China; xhf@njfu.edu.cn (H.X.); zhouhaiyanzj@njfu.edu.cn (H.Z.); zma@njfu.edu.cn (M.Z.); fancl@njfu.edu.cn (C.F.); yangyutu@njfu.edu.cn (Y.Y.); shuangxi@njfu.edu.cn (S.X.); 2 School of Agricultural Engineering, Jiangsu University, Zhenjiang 212013, China; zhangqingyi@ujs.edu.cn

**Keywords:** reinforcement learning, particleboard, deep learning, defect detection

## Abstract

Particleboard is an important forest product that can be reprocessed using wood processing by-products. This approach has the potential to achieve significant conservation of forest resources and contribute to the protection of forest ecology. Most current detection models require a significant number of tagged samples for training. However, with the advancement of industrial technology, the prevalence of surface defects in particleboard is decreasing, making the acquisition of sample data difficult and significantly limiting the effectiveness of model training. Deep reinforcement learning-based detection methods have been shown to exhibit strong generalization ability and sample utilization efficiency when the number of samples is limited. This paper focuses on the potential application of deep reinforcement learning in particleboard defect detection and proposes a novel detection method, PPOBoardNet, for the identification of five typical defects: dust spot, glue spot, scratch, sand leak and indentation. The proposed method is based on the proximal policy optimization (PPO) algorithm of the Actor-Critic framework, and defect detection is achieved by performing a series of scaling and translation operations on the mask. The method integrates the variable action space and the composite reward function and achieves the balanced optimization of different types of defect detection performance by adjusting the scaling and translation amplitude of the detection region. In addition, this paper proposes a state characterization strategy of multi-scale feature fusion, which integrates global features, local features and historical action sequences of the defect image and provides reliable guidance for action selection. On the particleboard defect dataset with limited images, PPOBoardNet achieves a mean average precision (mAP) of 79.0%, representing a 5.3% performance improvement over the YOLO series of optimal detection models. This result provides a novel technical approach to the challenge of defect detection with limited samples in the particleboard domain, with significant practical application value.

## 1. Introduction

Particleboard is defined as a practical engineered forest product that can be produced through the reuse of by-products in wood processing. Its popularization has been demonstrated to result in significant savings of forest resources and protection of the ecological environment. The manufacturing process of particleboard involves the hot-pressing of wood particles that are bonded with thermosetting resin adhesives to form an engineered composite panel [1]. The material’s excellent biodegradability has led to its widespread use in furniture manufacturing, architectural decoration, and industrial packaging [2]. However, surface defects such as scratches, indentations, and spots, which arise from machine errors and environmental interference during production, compromise the material’s esthetic quality and may also deteriorate its mechanical properties. In light of the ongoing escalation in particleboard production, propelled by the rapid growth of the customized furniture market [3], the establishment of an efficient and reliable quality inspection system [4] assumes paramount importance for ensuring the quality of particleboard products [5].

At present, the inspection of surface quality in the context of particleboard production is chiefly dependent on manual operations. Production line operators initiate the process by conducting preliminary screening through visual inspection combined with light reflection. This is followed by a detailed examination and grading of defects in dedicated manual inspection areas [6]. However, it has been demonstrated that visual fatigue resulting from prolonged working hours can lead to an increase in both false detection and miss detection rates [7]. Moreover, undetected surface defects have the potential to compromise the esthetic appearance of finished boards and may also result in structural issues during subsequent processing.

The advent of industrial automation technology has precipitated the gradual replacement of traditional manual operations with machine vision-based non-destructive inspection systems [8,9]. This transition has been accompanied by the demonstration of significant advantages in object detection [10,11] and instance segmentation [12,13,14] tasks. Nevertheless, conventional detectors necessitate a substantial number of region proposals, resulting in escalated training demands.

Recently, object detection methods based on deep reinforcement learning have demonstrated distinctive advantages. These approaches formulate object detection as a Markov decision process (MDP) [15,16], where a reinforcement learning agent iteratively optimizes bounding box adjustment strategies until reaching a terminal state for object localization [17]. Compared to traditional deep learning methods that require generating numerous candidate regions (typically tens of thousands), reinforcement learning approaches can accomplish detection tasks with significantly fewer candidate regions.

The present study aims to address the issue of the defective detection model’s suboptimal training outcomes when confronted with limited sample sizes. The paper explores the potential of reinforcement learning in the context of particleboard target detection, with the objective of proposing novel solutions to the associated challenges. The proximal policy optimization (PPO) algorithm introduces a gradient clipping mechanism to prevent excessive policy deviation and avoid overfitting with limited samples. Compared with value-based methods, PPO can generalize different combinations of actions through its policy network with lower sample dependency. Additionally, PPO adopts a stochastic exploration strategy that balances exploration and exploitation, enhancing its robustness. Most importantly, PPO achieves better sample efficiency than other methods, which is particularly crucial in small sample scenarios.

**Figure 1:**
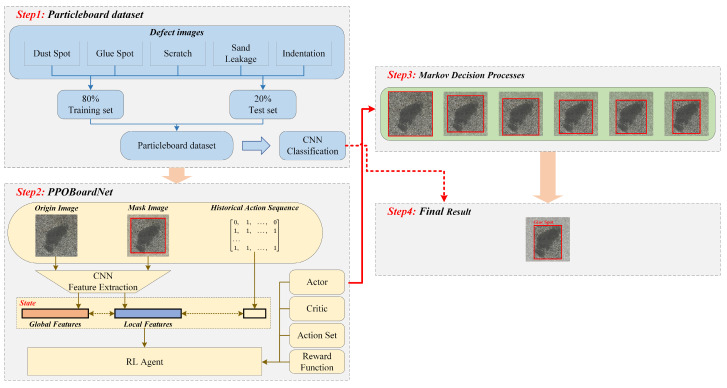
Workflow of PPOBoardNet algorithm.

This paper investigates an end-to-end reinforcement learning framework for object detection in particleboards, namely PPObordNet, based on PPO [18]. Unlike traditional supervised learning, which directly memorizes sample features, this algorithm focuses on learning a strategy for discovering anomalies. Through interaction with the environment, the model gradually acquires general principles for identifying abnormal regions in particleboard, rather than relying solely on the visual features of specific defects. The framework integrates variable discrete action spaces and feature extraction mechanisms using pretrained Convolutional Neural Networks (CNNs), as illustrated in Figure 1. Initially, when a particleboard image containing a single defect is input to the detection network, global features of the original image and dynamically updated local region features are extracted through a pretrained Residual Network (ResNet) model. These features are integrated with the action sequences from the previous four steps to construct comprehensive state representations. The reinforcement learning agent then randomly samples actions based on the current state to optimize bounding box positions, while the environment provides feedback according to predefined reward function. Through continuous policy optimization, the agent eventually triggers a termination action and completes object detection in conjunction with the classification network. This approach is significantly more efficient than traditional detection systems that require tens of thousands of candidate regions to complete detection tasks, as it requires only a few candidate regions (typically <10) to do so. This reduction in the number of regions is accompanied by a significant reduction in computational complexity and acceleration of model convergence.

The main contributions of this study can be summarized as follows:

(1) The development of a state representation enhancement module based on a pretrained ResNet model is a further advancement in the field. This module has been shown to significantly improve the decision-making capability of the agent by integrating global features, local features, and historical actions.

(2) The optimization of the policy network and value evaluation network architectures. The stacking of multiple linear modules enhances state feature extraction capability, accelerating the agent policy optimization and convergence process.

(3) The improvement of reward function design. A stage-wise main reward function based on IoU variation, combined with an auxiliary reward function based on bounding box differences, enhances the agent’s sensitivity to defects.

(4) The experimental results on a self-built particleboard defect detection dataset demonstrate that the proposed PPOBoardNet algorithm achieves detection performance comparable to YOLO.

## 2. Related Work

### 2.1. Large Vision Model

The evolution of object detection algorithms has been a subject of considerable research, with early region-based two-stage detectors, such as Region-CNN (R-CNN) [19], being superseded by single-stage detectors such as YOLO [20,21,22] and Single Shot MultiBox Detector (SSD) [23]. The former enhanced detection accuracy through collaborative optimization of region generation and classification, while the latter improved detection speed by directly regressing object location and category information. Hu et al. [24] improved the YOLOX network using multi-branch topology training combined with single-branch inference structure. By integrating the two-dimensional coordinates from RGB images with depth values, they constructed three-dimensional coordinates for apple picking points, achieving positioning errors of less than 7, 7, and 5 mm in the X, Y, and Z directions, respectively.

The employment of Transformer architecture in detection models [25], exemplified by the Detection Transformer (DETR) [26], has yielded a notable enhancement in detection performance through its incorporation of a global attention mechanism. A recent comprehensive study evaluated the detection performance of current state-of-the-art object detection algorithms on the innovative RSUD20K dataset [27], which includes diverse road scenes under various weather conditions. In this evaluation, the YOLOv6 series and the Transformer-based RTMEDT achieved mAP scores of 73.7% and 65.4%, respectively.

### 2.2. Reinforcement Learning Model

Recent years have witnessed significant progress in the application of reinforcement learning to object detection. Caicedo et al. [28] proposed an active detection model based on Deep Q-learning Network (DQN), which achieved object localization through the iterative optimization of agent behavior strategies, attaining an mAP of 46.1%. Cao et al. [29] introduced a hierarchical reinforcement learning detection framework that optimized the search process through multi-scale action strategies. By focusing on high-throughput regions and progressively refining localization, their experiments demonstrated that most objects could be detected within three steps. In addressing the slow convergence issue in reinforcement learning, Li et al. [30] proposed a combination of reinforcement learning with apprenticeship learning, achieving dynamic region optimization through constrained bounding box search space. This resulted in a mean Intersection over Union (IoU) of 69.13% in specific category detection tasks. Wen et al. [31] proposed a Task-Risk Consistent Intelligent Detection Framework (TRC-ODF) that leverages deep reinforcement learning for remote sensing object detection. The task-risk consistency reward mechanism optimizes model performance without human intervention, demonstrating significant improvements on multiple datasets with mAP increases of 0.8–5.4. Yin et al. [32] developed an interpretable remote sensing image detection model combining gradient-weighted class activation mapping with reinforcement learning, achieving over 85% accuracy on public datasets through an optimized ResNet backbone and improved reward function.

In order to simulate human visual perception processes, Jie et al. [33] proposed a novel tree-structured reinforcement learning framework. This method adaptively selects actions based on current states and search paths, demonstrating performance comparable to Faster R-CNN in multi-scale object detection. Addressing the issue of insufficient granularity in discrete action spaces, Wu et al. [34] developed a reinforcement learning system with parameterized action space, enhancing region proposal quality through discrete/continuous action co-optimization, achieving 77.4% mAP on the test set. Fang et al. [35] proposed a policy network architecture based on Spatial Transformation Network and Transformer to address the miss detection issue in small object detection. This framework first performs coarse localization on low-resolution images, then jointly feeds low-resolution background regions and high-resolution target regions into the detector for precise localization.

## 3. Materials and Methods

### 3.1. Particleboard Dataset

The experimental data presented in this study were obtained from three-layer eco-friendly particleboard manufactured by China Suqian Daya Wood Co., Ltd., Suqian, China. The specimen dimensions were 1220 mm × 2440 mm × 18 mm (length × width × thickness). All defective samples were evaluated and classified according to industry standards by experienced professional quality control inspectors.

#### 3.1.1. Image Acquisition System

**Figure 2:**
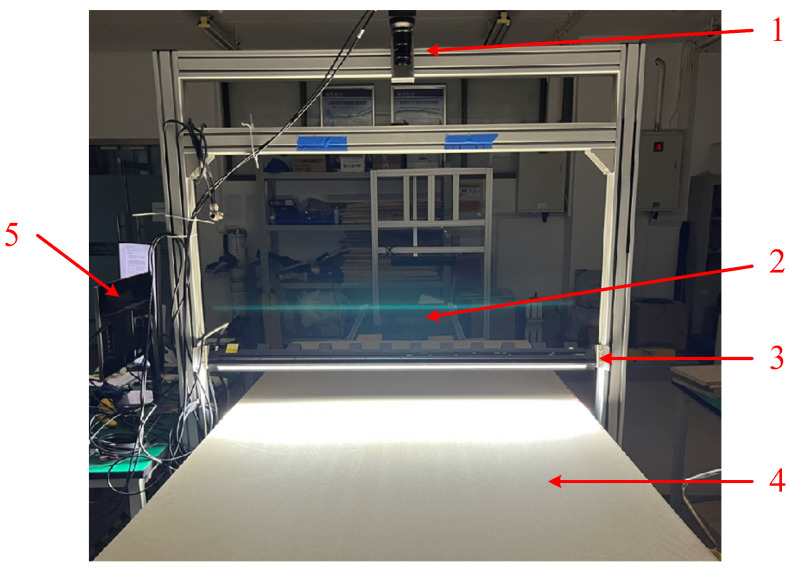
Image acquisition system. 1—Camera; 2—conveyor belt; 3—linear light source; 4—particleboard; 5—server.

The image acquisition system developed in this study is composed of several core components, including a line scan camera, a linear light source, a conveyor belt, and a data processing server (as shown in Figure 2). The specific model and technical parameters of the camera and lens are detailed in Table 1. The system employs a vertical configuration, maintaining a fixed working distance of 1.1 m between the camera lens and the particleboard surface. During the acquisition process, the particleboard moves at a constant speed on the conveyor belt, and the camera captures frames sequentially at pre-set line frequencies when triggered by the photoelectric sensor. The acquired data are transmitted to the server in real-time via Ethernet, where it is processed by an image stitching algorithm to generate complete RGB images of the particleboard.

#### 3.1.2. Dataset Construction

**Figure 3:**
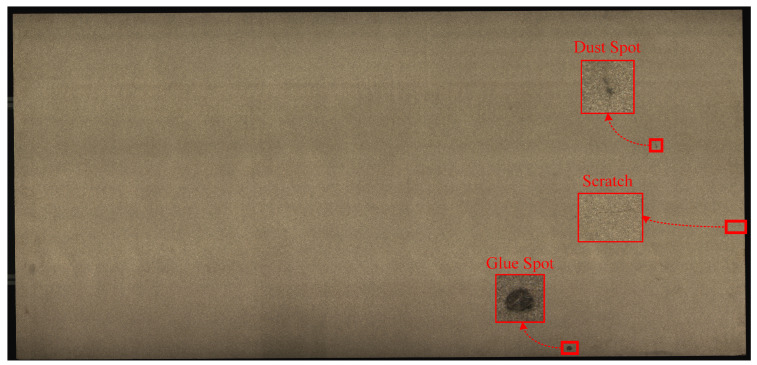
Original particleboard image.

The original particleboard image possesses a resolution of 8192 × 16,800 pixels, as illustrated in Figure 3. The raw images underwent a series of initial preprocessing steps, including background removal and other relevant operations. Thereafter, these images were subdivided into sub-blocks of 512 × 512 pixels. In conclusion, the image blocks containing defects were selected and annotated.

**Figure 4:**
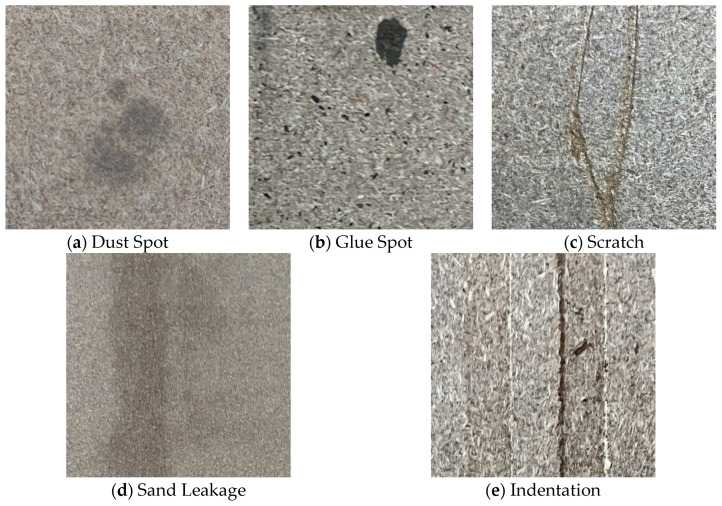
Schematic diagram of surface defects.

**Figure 5:**
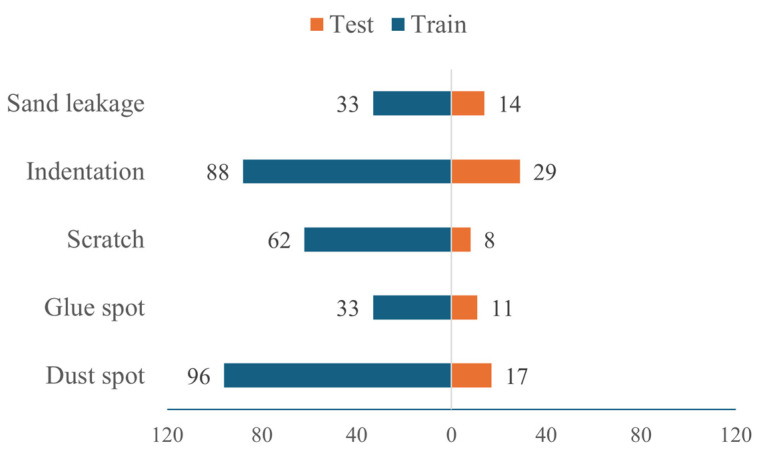
Distribution of defect samples.

This study encompasses five typical surface defects in particleboard: dust spot, glue spot, scratch, indentation, and sand leakage (as shown in Figure 4). Based on their formation mechanisms and scale characteristics, these surface defects are categorized as either small-scale or large-scale defects. Specifically, dust spots and glue spots, primarily caused by dust deposition in the production environment and uneven adhesive distribution, are classified as small-scale defects. Scratch, sand leakage, and indentation, primarily resulting from mechanical processing and logistics operations, are categorized as large-scale defects. A total of 391 valid defect samples were obtained through screening. Given that reinforcement learning algorithms require relatively few annotated samples, no data augmentation was performed in this study. The dataset was randomly partitioned into training and testing sets at an 8:2 ratio, with the specific distribution of each defect type illustrated in Figure 5.

### 3.2. Deep Reinforcement Learning-Driven Algorithm for Particleboard Defect Detection

PPO represents a state-of-the-art approach that introduces a policy clipping mechanism. This mechanism effectively constrains the policy update step size, thereby significantly reducing computational complexity. This reduction in complexity has led to the widespread application of PPO in large-scale sequential decision-making problems [36].

#### 3.2.1. Enhanced Actor-Critic Network Architecture

The high computational complexity and slow convergence rate of reinforcement learning algorithms have been identified as significant challenges. This paper proposes a Hierarchical Residual Module (HRM) as a solution to these issues. The HRM replaces convolutional operations with fully connected layers, thereby significantly reducing the model parameters. The core innovation of the HRM lies in the integration of Root Mean Square Normalization (RMSNorm) and ReLU Squared activation function. The former reduces computational overhead and enhances training stability, accelerating model convergence, while the latter enables effective capture of more complex feature relationships by enhancing the nonlinear representation capability of features.

**Figure 6:**
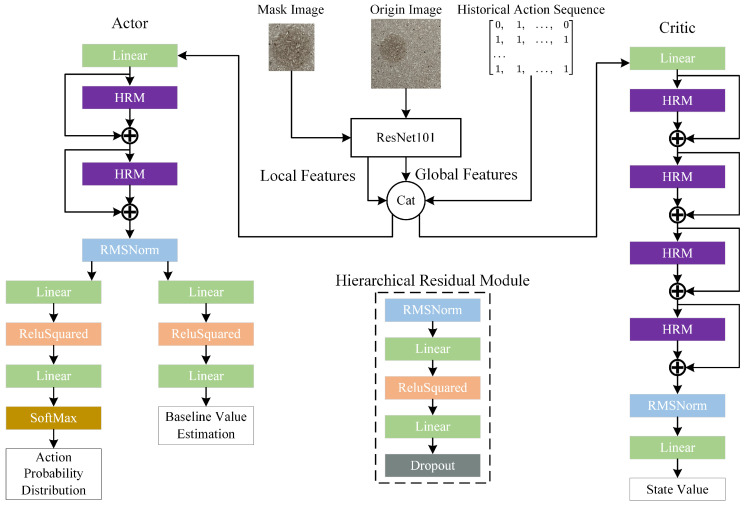
Architecture of input processing module.

In the proposed PPOBoardNet framework, the Actor network integrates HRMs with linear layers through residual connections to construct the backbone network. Two independent linear layer branches are utilized to generate action probability distribution and baseline value estimation, respectively. The Critic network is constructed through deep stacking of multiple HRM layers with residual connections, where its output state value function evaluates the accuracy of expected returns. This network architecture has been demonstrated to enhance the algorithm’s policy learning and state analysis capabilities, as illustrated in Figure 6.

#### 3.2.2. Enhanced State Representation Based on CNN

As demonstrated in Figure 6, the specific state combination method is illustrated. In order to enhance the state representation capability and optimize the agent’s action decision-making, this study employs a pre-trained ResNet101 model as the feature extractor.

Specifically, the original image and dynamically updated local images are input into the extractor separately to obtain global and local feature representations. These features are then integrated with the action sequence of the last four steps (one-hot vectors) into a one-dimensional feature vector, forming a complete environmental state representation. The multi-scale feature fusion strategy that has been adopted has been shown to significantly enhance the representation-al capacity of the state space, improve the agent’s target recognition capability, and provide certain directional guidance.

#### 3.2.3. Variable Action Space

**Figure 7:**
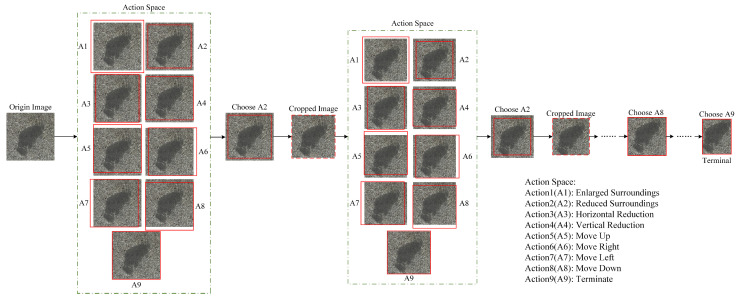
Sequential decision-making process for regional search by agent.

The action space delineated in this study encompasses three fundamental actions: scaling, translation, and termination. The magnitude of these actions is governed by an adjustable parameter, designated α, which is contingent upon the updated mask size. This variable action space design facilitates the model’s adaptation to detection tasks across diverse scales. Upon opting for the termination action, the agent signifies the successful localization of the current target, thereby prompting the immediate cessation of the iterative search process for that specific image. The reinforcement learning-based detection workflow is illustrated in Figure 7, where the action space design, characterized by its flexibility, has been shown to significantly enhance the model’s adaptability and detection efficiency.

#### 3.2.4. Composite Reward Function

The composite reward function integrates two key components: IoU gain and bounding box deviation penalty, providing immediate behavioral feedback for the agent. Specifically, the system assigns rewards based on dynamic changes in IoU, using preset multipliers or fixed values; meanwhile, when the detection box exceeds the valid region, penalty values are imposed to suppress unreasonable operations. When the termination action is selected, stepped rewards are given based on the IoU calculated from the previous action. This multi-dimensional reward mechanism effectively constrains the agent’s behavioral space and accelerates network convergence. The mathematical expressions for the composite reward and stepped reward are shown in Equations (1) and (2).

(1) $$r=\left\{\begin{array}{ll} \left(IoU_{i}-IoU_{i-1}\right)\times \beta_{u} & ,\;IoU_{i}-IoU_{i-1}>0\\ -r_{b} & ,\;IoU_{i}-IoU_{i-1}=0\\ -\left(IoU_{i}-IoU_{i-1}\right)\times \beta_{d} & ,\;IoU_{i}-IoU_{i-1}<0 \end{array}\right.,$$

(2) $$r=\left\{\begin{array}{ll} 30\cdot r_{b} & ,\;0.9\leq IoU_{i-1}\leq 1.0\\ 3\cdot r_{b} & ,\;0.8\leq IoU_{i-1}<0.9\\ r_{b} & ,\;0.7\leq IoU_{i-1}<0.8\\ -2\cdot r_{b} & ,\;0.6\leq IoU_{i-1}<0.7\\ -10\cdot r_{b} & ,\;0\leq IoU_{i-1}<0.6 \end{array}\right.,$$

where i denotes the action index (i=1,2,3…); $\beta_{u}$ and $\beta_{d}$ represent the weights for positive and negative rewards, respectively, and $r_{b}$ is the base reward value. Through calculation, the values of $\beta_{u}$, $\beta_{d}$, and $r_{b}$ are set to 10, 15, and 0.3, respectively.

### 3.3. Evaluation Metrics and System Specifications

In order to achieve an objective evaluation of the model’s performance, this study utilizes average precision (AP) and mAP as the primary evaluation metrics. Specifically, AP quantifies the precision of defect detection for each class, while mAP provides a comprehensive metric for the model’s overall detection performance. The detailed configurations of the experimental platform in terms of hardware and software are enumerated in Table 2.

## 4. Results and Discussion

### 4.1. Experiment Setting

All experiments in this section are conducted on the particleboard dataset described in Section 2.1. The experimental design consists of two main parts. Firstly, a parameter sensitivity analysis is performed to study the impact of action magnitude α on detection performance. Secondly, under the optimal parameter configuration, the proposed reinforcement learning detection model is compared with existing mainstream detection models to verify the feasibility and effectiveness of this method.

This study employs a ResNet101 model pre-trained on ImageNet [37] as the feature extractor, with the following main experimental parameters: maximum action steps per image is set to 20, learning rate is 0.0001, experience replay buffer capacity is 4000, and batch size is 64. The model undergoes training for a total of 4500 epochs and employs a combination of the ADOPT optimizer [38] and the Exponential Moving Average (EMA) technique, thereby enhancing the agent’s training stability and convergence speed.

### 4.2. Action Magnitude Parameter Optimization

**Figure 8:**
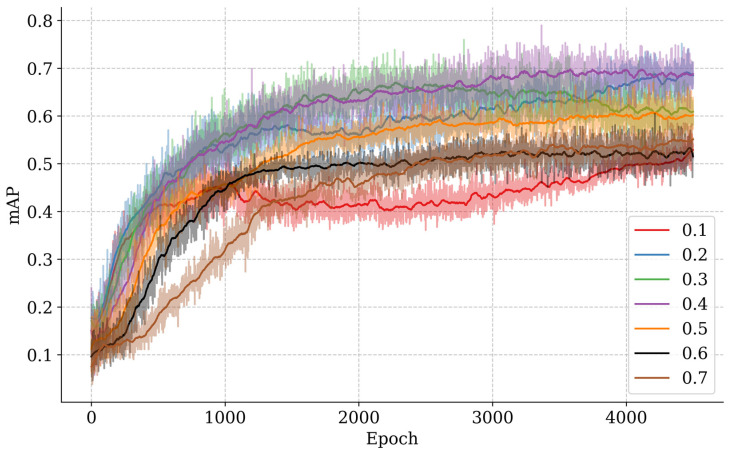
Smoothed mAP curves.

In the PPOBoardNet algorithm, the action magnitude parameter α has been shown to have a significant impact on detection performance. Excessive α values have been found to result in overly large boundary box update steps, which has been shown to increase the risk of missed detections, particularly in the case of small targets. Conversely, insufficient α values have been shown to reduce the boundary box update efficiency, significantly affecting detection speed. To investigate the parameter’s influence, this study conducted a parameter sensitivity analysis on the particleboard test dataset, systematically evaluating the impact of different α values on detection performance. Figure 8 shows the smoothed trends of mAP over the training progress on the test dataset.

The experimental findings demonstrate that the impact of the α value on the mAP exhibits notable phase characteristics during the training of the model. In the initial training stage (1–1000 epochs), the experimental data reveals a discernible negative correlation between the α value and the mAP. This phenomenon can be ascribed to the agent’s primary emphasis on task exploration during this stage, where larger action steps are disadvantageous for establishing stable state-action mapping relationships.

As the training progresses into the middle stage (1000–3000 epochs), the model gradually develops an understanding of the task, and the characteristics of different α values’ impact on performance begin to emerge. Specifically, when α = 0.1, the small boundary box update steps significantly reduce convergence speed and make it difficult to achieve precise localization of small-scale defects (e.g., dust spots and glue spots) within limited iterations, while increasing the risk of falling into local optima. Conversely, when α takes larger values (0.6~0.7), although the model can quickly locate large-scale defects (e.g., scratches, sand leakage, and indentations), the coarse-grained action space significantly reduces detection sensitivity for small-scale defects, leading to slower mAP growth. Experiments demonstrate that medium-range α values (0.2~0.5) attain an optimal balance in action granularity, thereby enabling the agent to sustain adequate exploration capability while concurrently exhibiting precise localization ability. This, in turn, results in the continuous optimization of model parameters under the objective of reward maximization and the attainment of stable enhancement in detection performance.

In the final training stage (3000–4500 epochs), the small-step strategy (α = 0.1~0.2) demonstrates an upward trend, while other parameter configurations appear to stabilize. The small-step model gradually mastered the action combination strategy, organically combining multiple actions to achieve more complex bounding box optimization.

As illustrated in Table 3, a quantitative analysis was conducted to investigate the impact of action step parameter α on the detection performance of PPOBoardNet. The experimental results demonstrated that extreme α values (α = 0.1 or 0.6~0.7) resulted in a substantial imbalance in the model’s detection capability across different scale defects, leading to an overall mAP below 60.5%, which is considerably lower compared to other parameter configurations.

The scale and visual characteristics of different defect types have been shown to significantly influence the performance of the PPOBoardNet defect detection algorithm. For defects of a smaller scale, such as dust spots and glue spots, the low pixel proportion in images and the ambiguous nature of their boundaries result in multiple iteration steps being required for accurate localization. This results in optimal detection accuracy of 66.7%. Conversely, large-scale defects, such as scratches and indentations, with their distinct geometric features and larger pixel proportion, can be localized within fewer iterations, achieving optimal detection accuracy of 100.0%. Sand leakage, a defect that occurs at a macro-scale, exhibits minimal average difference in color and texture features from normal board material, resulting in lower optimal detection accuracy (77.4%) compared to other large-scale defects.

The findings indicate that configurations within the α range of [0.2, 0.4] exhibit superior detection performance, with mAP values consistently surpassing 75.0%. Specifically, at α = 0.2, the model demonstrates enhanced bounding box refinement capability, attaining an optimal AP value of 66.7% in dust spot detection. Furthermore, the configuration with α = 0.3 demonstrates outstanding performance in large-scale defect detection, reaching AP values of 77.4% and 100.0% for the sand leakage and indentation categories, respectively.

However, the configuration with α = 0.4 achieves optimal balance in detecting all defect types, with the overall mAP reaching 79.0%, which is significantly higher than that of other configurations. For glue spot detection, the AP value reaches 66.7%, showing an improvement. For glue spot detection, the AP value reaches 66.7%, showing an improvement from 11.1% to 44.5% compared to other configurations; for scratch detection, the AP value reaches 100.0%, representing an increase from 14.3% to 42.9% over other configurations.

### 4.3. Performance Comparison

In order to verify the practical feasibility of the PPOBoardNet algorithm, this section conducts a comparative analysis with the mainstream object detection model YOLOv11 under optimal parameter configuration. In the comparative experiment, YOLOv11 series employs an input size of 640 × 640, batch size of 32, and SGD optimizer, with only model volume adjusted during the 500 epoch training process. The AP per category and mAP of each algorithm on the particleboard test dataset are presented in Table 4.

The experimental findings demonstrate that PPOBoardNet exhibits a substantial enhancement in performance when compared to existing methodologies. The mAP value of PPOBoardNet is 5.3% higher than that of the leading YOLOv11l method, attaining 100.0% and 92.9% accuracy in the domains of scratch and indentation detection, respectively. In addition, PPOBoardNet, operating on the basis of a Markov decision process, generates a single prediction box at a time, thereby significantly simplifying the subsequent post-processing workflow. By contrast, YOLO-based methods necessitate the implementation of complex Non-Maximum Suppression (NMS) processing to filter multiple candidate boxes, thereby increasing computational overhead.

Specifically, the detection time of YOLO series models increases significantly with the number of parameters, reflecting a direct relationship between model complexity and computational cost. The YOLOv11n model with the fewest parameters requires 60.16 ms to complete one detection, while the YOLOv11l model with the most parameters take 91.76 ms, showing a time increase of nearly 52%. This computational burden is particularly critical in online particleboard inspection systems with high real-time requirements, especially when conveyor systems operate at speeds exceeding 1 m/s, necessitating reduced detection time to allow sufficient reaction time for subsequent board screening processes. In contrast, our proposed PPOBoardNet model fully utilizes the efficient state transition characteristics of the Markov decision process, significantly optimizing forward propagation efficiency by reducing redundant computational paths. Furthermore, its innovative multi-scale information fusion mechanism employs a lightweight feature extraction network and multi-information feature aggregation strategy, substantially reducing computational complexity while ensuring detection accuracy. Under the same hardware environment (NVIDIA RTX 4090 GPU), PPOBoardNet requires only 36.05 ms to complete a full detection, reducing detection time from 40.08% to 60.71% compared to YOLO series models. This significant speed improvement not only enhances the system’s real-time response capability but also reduces deployment costs and energy consumption, providing a more feasible solution for resource-constrained terminal devices. Notably, PPOBoardNet maintains a high detection accuracy of 79.0% while keeping a lower detection time, demonstrating its superiority in balancing speed and accuracy. In the specific context of single defect detection on particleboard, the reinforcement learning-based method exhibits clear performance advantages over traditional CNN approaches.

**Figure 9:**
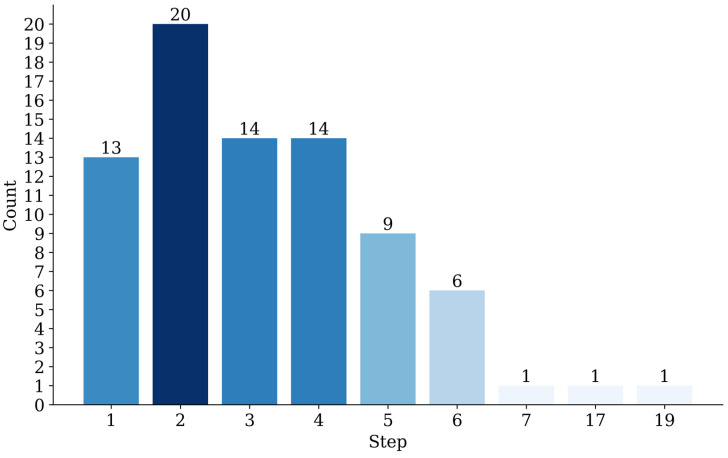
Detection step distribution of PPOBoardNet.

As demonstrated in Figure 9, the statistical outcomes of the detection steps for the PPOBoardNet algorithm are indicative of a high level of efficiency. The analysis of the particleboard dataset reveals that 88.6% of defect samples can be detected within five steps, with an average detection step count of 3.5, which is significantly lower than that of the YOLO series. Large-scale defects, such as sand leakage, indentation, and scratch, typically require 1–3 steps for detection, while small-scale defects, such as glue spot and dust spot, necessitate 2–5 steps. This validates the algorithm’s efficiency in detecting objects of different scales.

**Figure 10:**
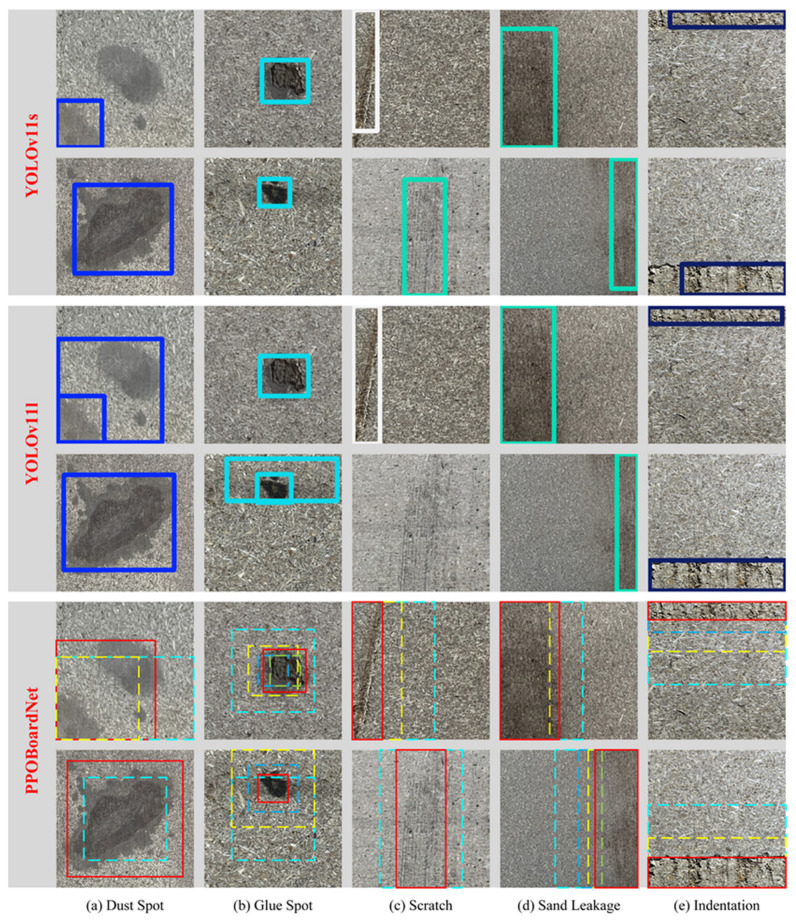
Visualization of Object Detection Results by PPOBoardNet Algorithm. In the results of PPOBoardNet, dashed boxes represent the localization process, while solid boxes indicate the final localization results. In the results of YOLO series, solid boxes of different colors represent different defect categories.

The detection results of the PPOBoardNet algorithm are presented in Figure 10. From the figure, it can be seen that YOLOv11l produces relatively accurate detection results for indentation and sand leakage, although there are still some false detections for dust spot and glue spot, and its performance in scratch detection is unsatisfactory, with some omissions. In contrast, YOLOv11s shows lower omissions across all categories, but its regression of bounding boxes is not precise enough, which is the main reason for its poorer detection performance. This discrepancy may be attributed to YOLOv11l’s larger number of parameters combined with a small dataset, leading to overfitting. Notably, PPOBoardNet achieves good detection results for various defect types without any omissions, and thanks to its action combination strategy, its fine control over bounding boxes surpasses that of the YOLO models.

## 5. Conclusions

In the context of forestry production and processing, particleboard represents a significant product. The advent of innovative production equipment has led to a decline in surface defects and a concomitant increase in the complexity of large-scale model training. This paper proposes PPOBoardNet, a defect detection system based on the Actor-Critic framework. The method incorporates a variable parameter action space and composite reward function, enhancing action flexibility and improving system feedback accuracy. Furthermore, the paper employs a multi-scale information fusion mechanism, integrating deep features from original and local images with historical actions into one-dimensional features, improving the state representation strategy.

The proposed PPOBoardNet algorithm has been shown to achieve 79.0% mAP on the particleboard dataset, representing a 5.3% performance enhancement over YOLO series detection models. The accuracy rates achieved for both scratch and indentation defect detection reach 100.0% and 92.9%, respectively. This finding provides a new technical pathway for particleboard defect detection, particularly in the advancement of few-shot defect detection technology.

Despite the favorable progress observed in the field of single object detection, certain limitations persist. On the one hand, an excessively small sample size can lead to poor generalization of the model. In future research, we will consider using data augmentation or knowledge distillation methods to expand the dataset or provide the model with certain prior knowledge. In addition, we will systematically evaluate the model’s robustness under different lighting conditions and camera angles. On the other hand, future research will concentrate on the development of parallel deep reinforcement learning algorithms with the aim of addressing multiple object defect detection in particleboard. In addition, further improvements will be made to the detection efficiency of micro-defects under few-shot conditions.

## Figures and Tables

**Table 1:** Detailed Specifications of Camera and Lens.

Equipment	Manufacturer	Technical Parameters	
MV-CL086-91GC	China Hangzhou Hikrobot Technology Co., Ltd., Hangzhou, China	Resolution	8192 × 6 pixels
		Line Frequency	4.7 kHz
LD21S01	China Jiangxi Phoenix Optical Co., Ltd., Shangrao, China	Focal Length	35 mm
		Aperture	F2.8-16

**Table 2:** Hardware and software specifications.

Category	Item	Specification
Software	Ubuntu	22.04
	PyCharm	2024.2.4
	Python	3.10
	CUDA	12.6
	PyTorch	2.5.1
Hardware	CPU	United States Silicon Valley Intel Core i9-14900K
	GPU	NVIDIA RTX 4090 24 GB

**Table 3:** AP per category and mAP on particleboard test dataset under different α values.

α	Dust Spot	Glue Spot	Scratch	Sand Leakage	Indentation	mAP
0.1	61.1	33.3	57.1	58.1	71.4	56.2
0.2	**66.7**	55.6	85.7	67.7	100.0	75.1
0.3	61.1	55.6	85.7	**77.4**	**100.0**	76.0
0.4	61.1	**66.7**	**100.0**	74.2	92.9	**79.0**
0.5	61.1	44.4	85.7	58.1	85.7	67.0
0.6	50.0	33.3	71.4	54.8	92.9	60.5
0.7	44.4	22.2	71.4	64.5	92.9	59.1

Note: Bold in the table represents the optimal value for each category or indicator under multiple models.

**Table 4:** Performance comparison between PPOBoardNet and YOLOv11 with different model volumes.

Method	Dust Spot	Glue Spot	Scratch	Sand Leakage	Indentation	Regions	Time	mAP
YOLOv11n	54.7	**99.5**	34.2	75.7	58.8	8400	60.16 ms	73.7
YOLOv11s	55.2	87.7	16.7	58.3	58.2	8400	64.46 ms	64.5
YOLOv11m	66.2	93.8	57.7	**92.1**	76.6	8400	79.18 ms	73.1
YOLOv11l	**76.2**	84.1	42.6	76.6	77.4	8400	91.76 ms	73.7
PPOBoardNet (ours)	61.1	66.7	**100.0**	74.2	**92.9**	**4**	**36.05 ms**	**79.0**

Note: Bold in the table represents the optimal value for each category or indicator under multiple models.

## Data Availability

The data are not publicly available as this study is still in progress.
